# Systems models, phenomics and genomics: three pillars for developing high-yielding photosynthetically efficient crops

**DOI:** 10.1093/insilicoplants/diy003

**Published:** 2019-04-25

**Authors:** Tian-Gen Chang, Shuoqi Chang, Qing-Feng Song, Shahnaz Perveen, Xin-Guang Zhu

**Affiliations:** 1 National Key Laboratory of Plant Molecular Genetics, CAS Center for Excellence in Molecular Plant Sciences, Shanghai Institute of Plant Physiology and Ecology, Chinese Academy of Sciences, Shanghai 200031, China; 2 State Key Laboratory of Hybrid Rice, HHRRC, Changsha 410125, China

**Keywords:** Crop yield, genomics, HYPEC, model-guided breeding, phenomics, systems model

## Abstract

Recent years witnessed a stagnation in yield enhancement in major staple crops, which leads plant biologists and breeders to focus on an urgent challenge to dramatically increase crop yield to meet the growing food demand. Systems models have started to show their capacity in guiding crops improvement for greater biomass and grain yield production. Here we argue that systems models, phenomics and genomics combined are three pillars for the future breeding for high-yielding photosynthetically efficient crops (HYPEC). Briefly, systems models can be used to guide identification of breeding targets for a particular cultivar and define optimal physiological and architectural parameters for a particular crop to achieve high yield under defined environments. Phenomics can support collection of architectural, physiological, biochemical and molecular parameters in a high-throughput manner, which can be used to support both model validation and model parameterization. Genomic techniques can be used to accelerate crop breeding by enabling more efficient mapping between genotypic and phenotypic variation, and guide genome engineering or editing for model-designed traits. In this paper, we elaborate on these roles and how they can work synergistically to support future HYPEC breeding.

## Introduction

The continued increase in global population, climate change and improved economic status throughout the world create a great demand for increased food productivity. Previous studies suggest that production of major crops needs to double to meet the projected demand by the year 2050, which requires an increased speed of yield improvement compared to the historical trends (Ray *et al.* 2013). Unfortunately, this increasing demand is in direct contrast with the decreasing rate of major crops’ yield enhancement in recent years, including rice, wheat, barley, soybean and maize (Peng *et al.* 2009; Zhu *et al.* 2010; Ray *et al.* 2012; Rötter *et al.* 2015). Finding new methods to improve major crop’s productivity has become a key challenge for plant biologists and crop breeders.

With the rapid development of genomics in plant sciences, molecular breeding (i.e. marker-assisted selection and genetic engineering breeding; Rao *et al.* 2014) is contributing more than ever to advancements in crop improvement (Peleman and van der Voort 2003; Moose and Mumm 2008; Rao *et al.* 2014). In the past decades, much progress has been made by molecular breeding in developing crops with higher yield, biotic and abiotic stress resistance, and improved grain quality and plant physiology (Moose and Mumm 2008; Rao *et al.* 2014). However, grain yield, as a super complex trait, is determined by source, sink and flow, and their complex interaction with the environment. These components and their interactions impact crop yields in a highly non-linear manner (Chang and Zhu 2017). Modelling is the most efficient method to pinpoint the morphological and physiological parameter to improve and define the optimal values of these parameters. Although the recently proposed approach ‘breeding by rational design’, which pyramids tens of superior genes from different parents, has resulted in high-yield and superior-quality progenies (Qian *et al.* 2016; Zeng *et al.* 2017), a systematic mining and assembling of superior genes, especially those interacting genes, will likely yield even better results.

Given the number of processes affecting crop yield, including canopy photosynthesis, root hydraulic and absorption property, material assimilation, metabolism, transport and utilization/storage, and the extensive interactions between these processes, what is the realistic approach to use in current breeding programmes to systematically increase crop yield? Here we propose a framework for future crop breeding, where phenomics, genomics and systems models will be the three major pillars. While extensive reviews are available in literature for each of these components (e.g. Furbank and Tester 2011; Morrell *et al.* 2012; Zhu *et al.* 2016), we discuss them as integrative components required for the future breeding of high-yielding photosynthetically efficient crops (HYPEC). Specifically, we first review the challenges of traditional crop breeding in realizing HYPEC. Then, we review the roles of plant systems models in understanding of complex biological systems and engineering for better plants, from which we argue that systems models hold great potential in guiding future crop improvement, especially with the synergy of systems models with genomics and phenomics. Finally, we propose a framework of model-guided breeding for HYPEC based on an effective combination of phenomics, genomics and systems models.

### Bottlenecks to breed HYPEC

The stagnation in crop yield enhancement in farmers’ field in recent years suggests that the potential of traditional breeding efforts is rapidly diminishing (Long *et al.* 2015). Following are the major challenges facing current HYPEC breeding:

1. Lengthy breeding cycle: In a traditional breeding programme, when a particular trait needs to be incorporated into an existing crop line, a typical practice is to find another line with the desired trait, then crossing and backcrossing it with the existing line multiple times until the trait is fixed while the existing superior traits are retained in the new line. However, this procedure is usually very time-consuming, e.g. it took 14 years to breed the line Y58S (Deng 2005), the female parental line for China’s hybrid rice cultivar Y-Liang-You 900, which reached a yield of 15.4 t ha^−1^ on ~7 hm^2^ scale in 2014 (Li *et al.* 2014).
2. Difficulty of identifying breeding targets for current elite crop lines: The breeding targets here are referred to the most limiting parameters for further improvement of photosynthetic efficiency and crop yield. Traditional breeders empirically select such targets to improve based on their own breeding experience. Most of the time, they identify traits that show superior performance in other elite lines and combine them together, with the assumption that there will be additive effects of such ‘superior’ traits (Zeng *et al.* 2017). However, some notions about traits that were traditionally regarded as beneficial for crop yield might not be accurate. As an example, a recently theoretical study showed that decreasing, rather than increasing, leaf chlorophyll content can increase both photosynthetic light and nitrogen use efficiency (Song *et al.* 2017). Another example is although breeders tend to select crop lines with higher leaf area index, it was found that decreasing soybean leaf area would raise crop yields under global atmospheric change (Srinivasan *et al.* 2017).
3. Difficulty of characterizing ideotypes for a defined environment: As discussed in the Introduction section, during decades of breeding, a number of ideotypes for high yields have been proposed by breeders. However, the current concepts of ideotype either differ drastically among breeders, or are physiological and architectural traits that are difficult to describe or quantify. For instance, some rice breeders consider erect panicles should be a feature of HYPEC since it can benefit canopy air circulation and enable deeper penetration of light into the canopy at noon (Chen *et al.* 2000), while some believe that 15–40 % higher yield can be gained by bending and maintaining panicles in a lower position inside a canopy (Setter *et al.* 1996). Moreover, the parameters in the current definition of ideotype are still mostly constrained to morphological traits during the grain filling stage (Peng *et al.* 1994; Yuan 1998; Chen *et al.* 2000), whereas physiological parameters and vegetative growth dynamics are rarely utilized in the breeding programmes. Furthermore, most of the parameters are related to above-ground traits, whereas the detailed root traits are rarely described. Lastly, current methods used to define ideotypes require experimental data and can therefore not be used to define ideotypes for future climate conditions, such as under elevated CO_2_ or temperature or altered precipitation patterns (Li *et al.* 2015). These issues in current ideotype definitions can be traced back to the lack of quantitative framework which can help define the contribution of different morphological or physiological parameters, to crop yield formation.
4. Lack of knowledge regarding the quantitative genetic control of critical traits: A typical case is that although numerous theoretical studies have suggested a great advance can be made in crop yield improvement by increasing photosynthesis (Zhu *et al.* 2010; Gu *et al.* 2014), the genetic basis of leaf photosynthetic capacity under high/low light and temperature, and leaf photosynthesis response for fluctuating environmental conditions are largely unexplored and not incorporated into crop breeding (Long 2014). There are two reasons for this lack of knowledge. First, many traits are under a complex network of genetic control, including crop height, heading date, tillering and grain size (Xing and Zhang 2010; Zuo and Li 2014). Second, it is difficult to characterize desired features, including morphological traits and physiological features for both above-ground and below-ground tissues, for a large number of candidate lines throughout their growing seasons.

### Systems models and their roles in supporting plant science research

Plant systems modelling refers to using mathematical models to quantitatively represent, integrate and simulate different physical, biochemical and physiological processes at a cell, organ, plant, population or ecosystem level. Systems model was first introduced into plant science for evaluation of canopy photosynthesis (de Wit 1965). Since then, systems models have been used to support the study of complex and highly non-linear systems, such as photosynthesis, assimilates partitioning, plant morphogenesis and soil–plant–atmosphere interaction. For example, by mathematically modelling root anatomy, 3D architecture, growth and function, the systems model OPENSIMROOT can be used to estimate the resource costs of developmental and anatomical traits, understand different root branching response patterns to soil nutrients and study among-root competition for nutrients (Postma *et al.* 2017).

Also, systems models can guide identifying targets to engineer for desired traits in a complex system. Zhu *et al.* (2007) developed a kinetic model of photosynthesis in a typical C_3_ mesophyll cell by describing detailed metabolic processes of the Calvin Benson cycle, starch synthesis, photosynthetic carbon oxygenation pathway, triose-P export and sucrose synthesis. The optimal nitrogen partitioning pattern among enzymes was predicted given a fixed amount of total protein nitrogen, and the key enzymes which need to be overexpressed for a higher photosynthetic rate, such as Rubisco, sedoheptulose-1,7-bisphosphatase, fructose-1,6-bisphosphate aldolase and ADP-glucose pyrophosphorylase, were identified. Consistent with the prediction, overexpression of SBPase in tobacco led to increased photosynthetic rate and biomass production in the field (Rosenthal *et al.* 2011). Systems models were recently shown to be able to predict physiological and growth properties beyond photosynthetic metabolism. For instance, Chew *et al.* (2014) integrated four existing models describing different aspects of *Arabidopsis* growth and development to form a multi-scale framework model. By adjusting only a few parameters from the original models, the authors could quantitatively predict the metabolic, physiological and biomass dynamics during vegetative growth of different *Arabidopsis* accessions grown under different environmental regimes. They found that by only adjusting one model parameter, the framework model generated significantly different phenotype on leaf number, leaf size, leaf size distribution and biomass at flowering, which was further validated by transgenic plants overexpressing miR156. This successful attempt suggests that it is a reachable goal now to use systems models to design and engineer plant phenotypes from basic molecular and biochemical properties.

### How can systems models contribute to HYPEC breeding?

After decades of research, a number of crop systems models have been developed, e.g. APSIM (McCown *et al.* 1996), CROPGRO (Boote *et al.* 1998) and DSSAT (Jones *et al.* 2003), and used to guide agronomical practices. Here we review how systems models contributed to crop breeding. Firstly, systems models can be used to predict crop yield. For instance, using an ensemble of 13 crop models, Li *et al.* (2015) predicted rice grain yields for multi-year experimental yield data at four sites with different environmental conditions with an uncertainty of <10 %. Secondly, systems models can help design cultivation practices. Recently, by using a 3D canopy photosynthesis model, Wang *et al.* (2017) designed a dual row planting scheme with asymmetric spacing of rows, which can decrease damage to plants and soil structure from harvest equipment with relatively little impact on canopy photosynthesis. Thirdly, systems models can guide marker-assisted selection with combination of quantitative trait loci (QTL) mapping. Gu *et al.* (2014) analysed the impact of genetic variation in leaf photosynthetic rate via QTL on crop biomass production using a mechanistic crop growth model GECROS in a rice introgression line population. This work highlights the potential of increasing leaf photosynthesis and crop production in rice (Long 2014).

### Challenges of crop systems models in guiding HYPEC breeding

To date, crop systems models have not been effectively utilized to guide HYPEC breeding, which is in contrast to the success of using leaf or canopy scale photosynthesis models in guiding crop improvements, e.g. accelerating recovery from photoprotection increased tobacco biomass (Kromdijk *et al.* 2016) as indicated by the theoretical prediction using a canopy photosynthesis model (Zhu *et al.* 2004). This lack of success in using crop systems models in guiding crop breeding is mainly due to the presence of several major barriers, which are as follows.

1. Lack of a comprehensive, multi-scale and mechanistic model describing biogenesis, function, growth and senescence of each organ in a plant growing in a community in the field: This is largely because crop yield is a complex trait. Developing a systems model covering each aspect of plant development mechanistically is far beyond the capacity of any one single research group. To tackle this challenge, a global consortium with the goal of developing such a model has been advocated (Zhu *et al.* 2016; Marshall-Colon *et al.* 2017).
2. Difficulty in model parameterization: After model integration, the first key step of applying the framework model to a specific crop cultivar grown under a certain environment is to assign a value for each model parameter in the crop systems model. However, considering that a complete crop systems model needs to simulate crop functional and structural dynamics during a whole growth season, plant–environment interaction and even genetic and transcriptional regulatory network dynamics, its thorough parameterization is, if not impossible, laborious and expensive. This greatly limits the application of systems models in crop breeding, where thousands of candidate plants need to be evaluated to identify superior lines.
3. Lack of information linking genomic and environmental information to model parameters: In the post-genomics era, molecular marker-based selection, genomic selection and genomic editing have become progressively faster and more efficient. Crop models can support crop molecular breeding if they can predict differences in the performance of different genotypes under different conditions (Yin *et al.* 2003b; Cooper *et al.* 2009). However, so far, the linkage between crop systems models and genomic information is still largely missing. Only a few biological processes have been modelled based on genomic information (Wilczek *et al.* 2009), while most of biological processes are still modelled based on macroscopic measurements, due to a lack of information on the molecular basis for these processes and the complex interaction between these processes and the environment.

### An emerging opportunity for achieving HYPEC with the support of crop systems models, phenomics and genomics

**Figure 1:**
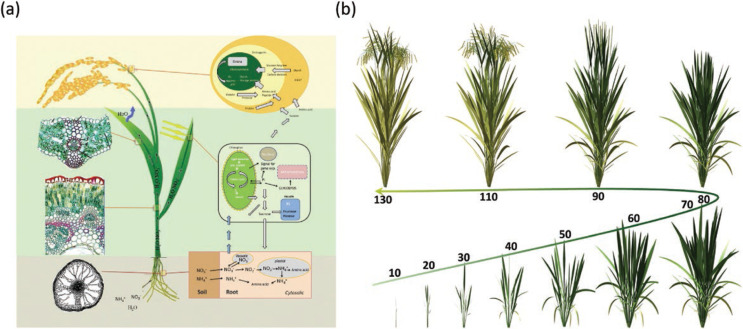
Elements needed for a mechanistic systems model of crop development and growth. (A) The structural (left) and functional (right) module. The structural module includes 3D modelling of each organ, the assembly of a whole plant and plant community, the micro-environment in the canopy and at the rhizosphere; the functional module includes carbon, nutrients and water uptake, assimilation and conversion in each organ, material transport within the xylem/phloem transport system and partitioning, plant–atmosphere interaction and root–soil interaction, organ senescence and material remobilization. (B) The changes of rice architecture during its life cycle. Detailed description can be found in the main text.

***Components needed to develop a comprehensive and mechanistic crop systems model.*** A mechanistic systems model of crop development and growth should be comprised of both structural and functional modules (Fig. 1). The structural module includes 3D modelling of development and growth of each organ, the assembly of a whole plant and plant community, the micro-environment in the canopy and rhizosphere. The functional module includes carbon, nutrients and water uptake and conversion in each organ, material transport in the xylem/phloem transport system and partitioning within the plant, plant–atmosphere interaction, root–soil interaction, organ senescence and material remobilization (Fig. 1A, B). To date, a number of elaborate systems models comprising the two modules have already been developed. Here we summarize the minimal set of models required to form a prototype plant-community level systems model. Firstly, modelling of organ morphogenesis must be included. Both above- and under-ground organ development can be modelled using L-system or analogues to generate and store branching structures and their relationships (Lynch *et al.* 1997; Fournier and Andrieu 1999; Watanabe *et al.* 2005; Leitner *et al.* 2010). Secondly, modelling of organ growth for the formed organs is required. Currently, the growth of organs is modelled based on empirical growth pattern extracted from experimental observations (Yin *et al.* 2003a). However, to enable models to predict growth under different environments and even future climates, mechanistic and quantitative models incorporating regulatory modules for the regulation of cell division and expansion are urgently needed and are possible now (e.g. the mechanistic modelling of cell division and growth on leaf growth; Fox *et al.* 2018). Thirdly, modelling of CO_2_ and nutrients (e.g. nitrogen, phosphate and potassium) assimilation is needed. The 3D architecture-based canopy and root models, using organ physical and physiological properties, micro-environment around plants and inside organs as input, can predict canopy photosynthesis and root absorption activities (Song *et al.* 2013; Xiao *et al.* 2016; Postma *et al.* 2017). Followed by photosynthetic CO_2_ uptake, modelling of assimilates transport, partitioning and potential remobilization (Allen *et al.* 2005; Yin and van Laar 2005; Thorpe *et al.* 2011; Chang and Zhu 2018) is also required. Finally, the available assimilates in different organs will drive the next round of organ growth and hence needs to be simulated in a complete plant-community level systems model as well.

Integration of these individual models will not be an easy task as they usually have different time steps, different types of input/output data, work on different temporal and spatial scales, and were developed using different programming languages. The key challenge now is to begin a community effort to develop a universal interface to integrate them effectively (Zhu *et al.* 2016). Some progress has already been made such as initial attempts of multi-scale model integration in *Arabidopsis* and maize (Chew *et al.* 2014; Postma *et al.* 2017). Moreover, a number of programmes have been set up with the goal of reconstructing a functioning crop plant and community of plants from the genes upward by effective model development and integration (Marshall-Colon *et al.* 2017; Xiao *et al*. 2017).

***High-throughput phenotyping enables morphological and physiological parameter quantification to support model parameterization and application.*** To guide crop breeding, crop systems models need to be well validated and further parameterized for the target crops or particular cultivars of a crop. This is a major challenge due to the difficulty of collecting comprehensive structural and functional parameters needed for crop systems models, as discussed above. For parameterization of a model simulating a simpler system, empirical relationships derived from fitting existing data can be used to convert measurable traits into model parameters. However, for an irreducible complex system, inverse problem theory needs to be applied to enable mapping from measurable traits to model parameters (Tarantola 1987).

High-throughput phenotyping technology now enables researchers to collect an unprecedented quantity and quality of phenomic (i.e. morphological, physiological, biochemical and molecular trait) data for plants grown at different temporal and spatial scales and environmental conditions (Table 1). Many morphological traits previously measured manually, such as plant height and biomass, days to heading, panicle and grain features, can now be measured more rapidly and accurately with phenomics techniques (Crimmins and Crimmins 2008; Tilly *et al.* 2014; Yang *et al.* 2014). Phenomics also enables the measurement of morphological traits that are difficult to track visually and non-destructively. For example, magnetic resonance imaging (MRI) and X-ray computed tomography (CT) can be used to quantify 3D root morphological parameters *in situ* in soil (Metzner *et al.* 2015). Many physiological traits, which historically can only be measured manually, can also be measured with phenomics platforms. Yang *et al.* (2014) developed an automated high-throughput rice traits scorer, which can quantify grain number per panicle, grain shape, filled-grain ratio and 1000-grain weight. Dreccer *et al.* (2014) developed a high-throughput approach to quantify wheat stem non-structural carbohydrates (NSC) in the field using hyperspectral reflectance. Later, Wang *et al.* (2016) proposed a more robust approach based on infrared spectrum scanner for evaluation of stem NSC in rice. Likewise, leaf nitrogen concentration, leaf mass per area, maximum rates of RuBP carboxylation and regeneration can be measured using fresh-leaf reflectance spectroscopy (Serbin *et al.* 2012). High-throughput molecular level data collection is now *et al.* (2016) possible, as demonstrated by the high-throughput transcriptome sequencing and the emerging robotized enzymes activity measurement platforms (Gibon *et al.* 2004; Tombuloglu *et al.* 2015). A comprehensive list of these parameters that need to be collected at different developmental stages of crop growth and development and corresponding state-of-the-art phenotyping methods are summarized in Table 1.

**Table 1:** Key parameters used in model-guided crop breeding by design and corresponding state-of-the-art phenotyping methods.

Parameter	Phenotyping method	Throughput/accuracy	Reference
Environmental parameters during the whole growth season			
Direct/scatter light intensity	Field weather station	High/high	–
Air property	Field weather station	High/high	–
Soil property	Field soil sensors	High/high	–
Whole-plant features at different growth stages			
Days to heading/harvest	Field repeat digital imaging	High/high	Crimmins and Crimmins (2008)
Canopy microclimate and photo-synthetic parameters	Field canopy photosynthesis and spiration measurement system	Medium/high	Song *et al.* (2016)
3D canopy structure	Stereovision	Medium/high	Hui *et al.* (2018)
	Parameterization from 2D images	Medium/high	Song *et al.* (2013)
Organ optical property	Full-spectrum all-direction organ reflectance and transmittance meter	Medium/high	In development
Organ anatomy structure	Nuclear magnetic resonance or microcomputed tomography	Low/high	Brodersen and Roddy (2016)
	3D imaging	Low/high	Palmer *et al.* (2015)
Phloem/xylem (un)loading kinetics	–	–	–
Phloem/xylem hydraulic conductance	–	–	–
Leaf features at different growth stages			
Activity of photosystems	Kinetic chlorophyll fluorescence im- aging systems	High/high	Tschiersch *et al.* (2017)
Stomatal conductance	Thermal infrared imaging	High/medium	Jones (1999)
	Gas exchange	Medium/high	Juenger *et al.* (2005)
Nitrogen content	Leaf reflectance spectroradiometer	High/high	Yendrek *et al.* (2016)
Enzymes activity of basic carbon and nitrogen metabolism	Robotic enzyme activity platform	High/High	Gibon *et al.* (2004)
Hydraulic conductance	Evaporative flux method, rehydration kinetics methods, etc.	Medium/medium	Flexas *et al.* (2013)
Stem features during grain filling			
Strength	In-lab prostrate tester	Low/high	Kashiwagi and Ishimaru (2004)
Carbohydrates storage	In-lab near-infrared spectrum scanner	Medium/high	Wang *et al.* (2016)
	Field canopy hyperspectral reflectance	High/medium	Dreccer *et al.* (2014)
Carbohydrates metabolic activity	Robotic enzyme activity platform	High/high	Gibon *et al.* (2004)
Panicle features during grain filling			
Grain number per panicle	In-lab 2D imaging	Medium/high	Yang *et al.* (2014)
Panicle architecture	In-lab 2D imaging	Medium/high	Crowell *et al.* (2014)
Photosynthesis and respiration	Gas exchange	Low/high	In development
Endosperm cell division activity	–	–	–
Endosperm cell energy metabolism and starch/protein synthesis activity	Robotic enzyme activity platform	High/high	Gibon *et al.* (2004)
Filling pattern for grains at different panicle position	Manual measurement	Low/high	Yang *et al.* (2005)
Filled-grain ratio, grain shape, grain weight	In-lab machine vision-based facility	High/high	Yang *et al.* (2014)
Root features at different growth stages			
Growth activity	–	–	–
Minerals absorption activity	Robotic enzyme activity platform	High/high	Gibon *et al.* (2004)
Hydraulic conductance	Evaporative flux method	Low/high	Sack and Scoffoni (2012)
	Rehydration kinetics methods	High/medium	Brodribb and Holbrook (2003)
Amount and composition of exudates	High-performance liquid chromatography or gas chromatography/mass spectrometry	Low/high	Li *et al.* (2013)
3D architecture	X-ray computer tomography	Medium/medium	Pfeifer *et al.* (2015)
	Magnetic resonance imaging	Low/medium	van Dusschoten *et al.* (2016)

It is worth emphasizing here that systems models may also drive the development and expansion of phenotyping tools. Firstly, systems models can be used to identify critical morphological and physiological parameters to be screened using phenomic approaches. As an example, a model of carbon isotope discrimination and stomatal conductance suggested that leaf water use efficiency can be ‘recorded’ in the ^13^C/^12^C ratio of the leaf (Condon *et al.* 2002). This ratio was used to guide the development of *Drysdale*, a bread wheat cultivar with improved transpiration efficiency and grain yield (Condon *et al.* 2004). Furthermore, systems models can be used to integrate different directly measured parameters to calculate or predict new and unmeasurable parameters that represent status of growth and development, e.g. through the integration of 3D plant architecture data and leaf optical properties, canopy photosynthesis models can predict the total canopy photosynthetic CO_2_ uptake rate (Song *et al.* 2013), which is a critical physiological parameter related to HYPEC breeding. Lastly, models of plant morphology or physiology can support parameter extraction from raw phenotype data by generating large amounts of synthetic data as a training set, which was exemplified recently by using synthetic plant architecture in deep learning to enable accurate leaf counting in photographs of rosette plants (Ubbens *et al.* 2018).

***Genomics accelerates the development of crop systems models and realization of crops designed with these models.*** As mentioned above, a key feature of future advanced crop systems models is to predict phenotype from genotype. How can one incorporate genomic information into systems models? The first approach is to construct mapping functions between molecular markers and macroscopic model parameters. For example, heading time of individual lines in a recombination inbred population was successfully predicted under different environments based on the parameterization of four model parameters from corresponding QTL markers (Yin *et al.* 2005). Similarly, grain number per panicle of rice was predicted for ~1500 elite hybrid rice lines based on the number of superior alleles in each line (Huang *et al.* 2015). Recently, human height was accurately predicted from genotype data in a large population using a machine learning approach (Lello *et al.* 2018); this approach should also be applicable in the prediction of crop complex traits. Constructing a genetic regulatory network to directly predict phenotype from genotype is another approach for model parameterization. By using a gene network-based model, Wilczek *et al.* (2009) accurately predicted flowering time of different genotypes of *Arabidopsis*. Furthermore, with the genome information of *Mycoplasma genitalium*, a whole-cell computational model was constructed to predict a wide range of phenotypes from its genotype (Karr *et al.* 2012). It is foreseeable that these different approaches will be used either individually or together to obtain model parameterization with progressively improved accuracy.

Genomics can accelerate identification of alleles or QTLs controlling traits identified by crops systems model as targets to produce HYPEC. Such QTLs or alleles have already been used in molecular marker-assisted breeding (Collard *et al.* 2005). Selection based on molecular markers outperforms traditional selection based on phenotype which can be influenced by growth environments, and has been widely used in breeding crops for biotic/abiotic resistance, high yield and superior quality, including maize (Ribaut and Ragot 2007; Prasanna *et al.* 2010), rice (Singh *et al.* 2001; Zhou *et al.* 2003; Jena and Mackill 2008), wheat (Gupta *et al.* 1999; Miedaner and Korzun 2012) and potato (Barone 2004). Genomic information is also required to support current genome editing techniques which can directly modify genomic sequences to modify particular traits or be used to perform multiple genes knock-ins, knock-downs or knock-outs simultaneously (Czarnecki *et al.* 2016; Zhu *et al.* 2017; Miao *et al.* 2018). Compared to marker-assisted breeding, breeding using genome editing techniques can accelerate production of HYPEC since it can potentially produce homozygous target gene(s) in one generation (Zhang *et al.* 2014). Finally, genomic information and the function map between model parameter and genotype will be used to guide gene or allele pyramiding (Zeng *et al.* 2017) or genome editing (Rodriguez-Leal *et al.* 2017) to realize the predicted crop ideotype.

### Synergy between systems models, phenomics and genomics for HYPEC breeding

**Figure 2:**
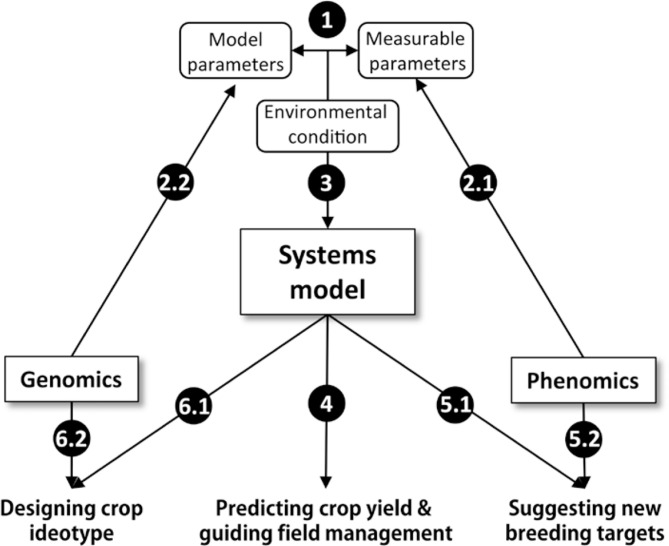
A conceptual framework of model-guided breeding for HYPEC with an effective combination of systems models, genomics and phenomics. 1, mapping between model parameters and experimentally measurable parameters; 2.1, phenomics facilitated quantification of measurable parameters; 2.2, direct prediction of model parameters from genome; 3, prediction of crop growth using a parameterized systems model; 4, prediction of crop yield and selection of optimal field management practice using mechanistic systems models; 5.1, systematic identification of breeding targets; 5.2, phenomics assisted screening of breeding targets; 6.1, defining of crop ideotype; 6.2, genomics assisted realization of crop ideotype.

Here we summarize the above contents and describe the framework of systems model-guided HYPEC breeding, which will be built on the synergism of systems models, phenomics and genomics (Fig. 2). Firstly, a mapping function between model parameters and experimentally measurable parameters is needed, either by model reduction or using inverse problem theory (item 1 in Fig. 2). Secondly, high-throughput phenotyping can provide required phenomics data for parameterization (item 2.1 in Fig. 2), and genomics can help predict model parameters from genomic sequences (item 2.2 in Fig. 2). Thirdly, the parameterized model will be calibrated and/or improved by simulating crop growth under different environmental conditions (item 3 in Fig. 2). Fourthly, a well-established and parameterized systems model can be used to predict crop yield for one specific crop line grown under particular environment, and to guide field management practices, e.g. choice of crop’s growth ecological zone, decision of water and fertilizer regimes and optimization of plant spacing (item 4 in Fig. 2). Such predictions can directly benefit farmers, in terms of achieving higher output or higher ratio of output/investment. Fifthly, systems models are used to identify new breeding targets. Specifically, by parameters perturbation and *in silico* simulation, crop systems models can be used to identify key parameters or parameter combinations to increase yield (item 5.1 in Fig. 2). These identified critical parameters can be screened through phenomics facility assisted trait measurements (item 5.2 in Fig. 2). Sixthly, crop systems models can be used together with optimization algorithms to design optimal combinations of plant structural and functional parameters, including physiological, biochemical and molecular parameters, during the whole growth season under defined environmental conditions to gain maximal productivity (item 6.1 in Fig. 2). Then, genomic information and technology will be used to realize the designed crop ideotype (item 6.2 in Fig. 2). Undoubtedly, this framework can only be developed and improved with the participation of scientists from systems biology, molecular biology, engineering science and computational science. During this process, a close cooperation with breeders is extremely important to ensure that the model will better capture the breeder’s experience in field managements, breeding targets identification or ideotype definition.

## Conclusion

Improving photosynthetic efficiency over a whole growing season is now recognized as a major option to drastically improve crop yield, for which all the sink and flow related processes also need to be simultaneously considered to achieve the expected gain in crop yield potential. In this aspect, many breeding organizations, such as CIMMYT, have recognized this and created physiology-based breeding programmes to simultaneously improve source-, sink- and flow-related traits (Reynolds and Langridge 2016). This work presents the rationale and a strategy to expedite HYPEC breeding by combining systems models, phenomics and genomics techniques in the same breeding programme. We envisage that effective combination of these different techniques, which are, at this point, mostly scattered in different research or breeding organizations, will create enormous synergy and help catalyse another round of rapid gain in crop yield potential in the coming decades.
